# Association between nutritional status and pneumonia in patients with spontaneous intracerebral hemorrhage

**DOI:** 10.3389/fnut.2025.1547655

**Published:** 2025-03-12

**Authors:** Xiaoyan Zhang, Lele Kang, Pianpian Du, Dongjuan Xu, Hongfei Li, Zhuangzhuang Jiang

**Affiliations:** Department of Neurology, Affiliated Dongyang Hospital of Wenzhou Medical University, Dongyang, China

**Keywords:** spontaneous intracerebral hemorrhage, stroke-associated pneumonia, nutritional risk screening-2002, controlling nutritional status, prognostic nutritional index, malnutrition

## Abstract

**Background:**

Stroke-associated pneumonia (SAP) is a common and serious complication in patients with spontaneous intracerebral hemorrhage (SICH), contributing to prolonged hospital stays and poor outcomes. Nutritional status has been linked to the development of SAP in patients with ischemic stroke, but its role in SICH patients remains understudied. This study aims to evaluate the predictive value of the Nutritional Risk Screening-2002 (NRS-2002) score for SAP in SICH patients and to compare it with other nutritional assessment tools.

**Methods:**

This retrospective observational study included 404 consecutive SICH patients admitted to Dongyang People’s Hospital from January 2023 to May 2024. Nutritional risk was assessed using the NRS-2002 score upon admission, and SAP was diagnosed within the first 7 days of hospitalization. Univariate and multivariate logistic regression analyses identified risk factors for SAP, and receiver operating characteristic (ROC) curves were used to compare the predictive accuracy of the NRS-2002, Controlling Nutritional Status (CONUT) score, and Prognostic Nutritional Index (PNI) for SAP.

**Results:**

Among the 404 patients, 97 developed SAP. A higher NRS-2002 score was significantly associated with an increased risk of SAP (OR: 1.575, 95% CI: 1.134–2.186, *p* = 0.007). ROC analysis showed that the NRS-2002 score (AUC: 0.768, 95% CI: 0.716–0.820) outperformed the CONUT (AUC: 0.597, 95% CI: 0.530–0.663) and PNI (AUC: 0.588, 95% CI: 0.519–0.657) in predicting SAP (*p* < 0.05). Subgroup analysis revealed that the NRS-2002 score ≥ 3 was particularly predictive of SAP in patients with weight loss, severe stroke, and those without hypertension or with diabetes.

**Conclusion:**

The NRS-2002 score is a valuable predictor of pneumonia in SICH patients, with higher scores correlating with a significantly increased risk of SAP. This highlights the importance of early nutritional assessment in identifying high-risk patients and potentially guiding clinical interventions to reduce SAP incidence.

## Introduction

Stroke-associated pneumonia (SAP), defined as lower respiratory tract infection occurring within 7 days in non-mechanically ventilated stroke patients (1), is a common and serious complication that significantly increases adverse outcomes and mortality (2, 3). Muscle weakness after a stroke impairs swallowing and coughing, raising the risk of aspiration of food, saliva, or liquids, which introduces pathogens into the lungs (4). Moreover, stroke-induced immunosuppression weakens the body’s immune defenses, further facilitating lung infections (5). SAP not only prolongs hospital stays and delays functional recovery but also increases the risk of complications such as deep vein thrombosis, gastrointestinal bleeding, and atrial fibrillation (6). As a major contributor to poor stroke outcomes, understanding its underlying mechanisms and identifying high-risk groups is essential.

Malnutrition affects approximately 6 to 62% of stroke patients admitted to hospitals (7, 8). Recent studies have demonstrated a close association between nutritional status and the development of SAP. Nutritional indicators, including the albumin-to-globulin ratio, blood lipid levels, Body Mass Index (BMI), and fibrinogen-to-prealbumin ratio, have been found to be associated with the development of stroke-associated pneumonia in ischemic stroke (IS) patients (9–11). Additionally, nutritional assessment tools, which are methods for evaluating a patient’s nutritional status based on a combination of clinical data, such as the Nutritional Risk Screening-2002 (NRS-2002), Prognostic Nutritional Index (PNI), Controlling Nutritional Status (CONUT), and Geriatric Nutritional Risk Index (GNRI) have been identified as useful predictors of SAP risk in IS patients (12–14). However, research on the relationship between nutritional status and pneumonia associated with spontaneous intracerebral hemorrhage (SICH) remains limited. Although both IS and SICH share common risk factors for developing pneumonia, there are also distinct risk factors specific to pneumonia following SICH. First, SICH patients are typically required to remain bedridden in the acute phase to minimize the risk of worsening bleeding (15), while IS patients are encouraged to mobilize early to reduce complications like venous thromboembolism and promote rehabilitation (16). Prolonged bed rest in SICH patients is a significant risk factor for the development of aspiration pneumonia (17). Second, consciousness impairment caused by brain edema after a stroke increases the risk of pneumonia (18). Brain edema is generally more severe in SICH than in IS (19), making imaging factors closely related to edema, such as lesion volume and location, especially important in SICH-associated pneumonia. Furthermore, hemorrhagic infiltration into the ventricles is a distinctive imaging feature in SICH patients. Finally, while cardiac factors, such as atrial fibrillation, play a significant role in pneumonia following IS (20), they have relatively less influence on pneumonia after SICH (21). Evidence suggests that patients with hemorrhagic stroke are more likely to develop pneumonia compared to those with IS (22). Therefore, it is crucial to explore the relationship between nutritional status and pneumonia in patients with SICH.

The NRS2002 score is a nutritional screening tool based on over 100 randomized controlled trials (RCTs) as its evidence base (23). Since its introduction in 2002, numerous studies have further validated its effectiveness in clinical practice (24, 25). Many clinical nutrition guidelines, such as those from the European Society for Parenteral and Enteral Nutrition (ESPEN) and the Chinese Society for Parenteral and Enteral Nutrition (CSPEN), recommend the NRS2002 as the preferred tool for nutritional risk screening. The NRS2002 score evaluates patients’ nutritional status based on three parameters: impaired nutritional status, disease severity, and age (23). The CONUT and PNI scores evaluate nutritional status from the perspective of immune and inflammatory responses. Since stroke induces immune and inflammatory reactions (26), these scores are particularly relevant for assessing the nutritional status of affected patients. Additionally, the parameters involved are readily available in routine clinical practice, ensuring they do not impose an additional burden on healthcare providers. This study aims to explore the relationship between the NRS-2002 and SICH-associated pneumonia. Additionally, it seeks to compare the predictive performance of the NRS-2002 score with other nutritional scoring systems, including the CONUT and the PNI.

## Materials and methods

### Patients

In this retrospective observational study, we focused solely on consecutive patients with spontaneous intracerebral hemorrhage admitted to Dongyang People’s Hospital from January 1, 2023, to May 31, 2024. The study received ethical approval from the Ethics Committee of Dongyang People’s Hospital (2024-YX-332) and was conducted in accordance with principles outlined in the Declaration of Helsinki. To safeguard patient privacy, all personal information was anonymized during data collection and analysis. SAP was diagnosed within the first 7 days of admission according to the criteria based on clinical and laboratory indices of respiratory tract infection (e.g., fever, cough, auscultatory respiratory crackles, new purulent sputum, or positive sputum culture), and supported by typical chest X-ray or chest computed tomography imaging (1). Patients were included based on the following criteria: (1) Aged 18 years or older. (2) Admission with a primary diagnosis of SICH, confirmed by cerebral computed tomography (CT) or magnetic resonance imaging (MRI). (3) Signed informed consent was obtained from the patients or their legally authorized representative. The exclusion criteria were: (1) Intracerebral hemorrhage resulting from brain tumors, aneurysms, arteriovenous malformations, cavernous hemangiomas, or traumatic brain injuries. (2) Patients who underwent mechanical ventilation. (3) Pre-existing pulmonary infection prior to admission. (4) Severe hepatic and renal insufficiency. (5) Hospitalization duration of less than 24 h. (6) Incomplete or missing data.

### Sample size calculation

We estimated the minimum sample size required for the study using PASS software. Due to the lack of data on the NRS-2002 and stroke-related pneumonia in patients with SICH, we referred to data from studies on NRS-2002 and stroke-associated infections in patients with IS (12). The expected odds ratio (OR) for exposure was 2.3, and the proportion of patients with nutritional risk in the non-pneumonia group was 29%. The significance level (*α*) was set at 0.05, and the power (*β*) was set at 0.80. The minimum sample size was calculated using the “two independent proportions” module of PASS software with a 1:1 group allocation.

### Data collection

We gathered a comprehensive set of baseline characteristics, including demographic data, vascular risk factors, comorbidities, laboratory results, and neuroimaging findings upon admission. Hypertension was identified by the prior use of antihypertensive medications (27). Diabetes mellitus was defined as the use of glucose-lowering medications or hemoglobin A1c ≥6.5% (28). Conditions such as ischemic heart disease (IHD), dementia, chronic obstructive pulmonary disease (COPD), and cancer were considered present if there was clear medical documentation or a confirmed diagnosis at discharge. A history of stroke was defined by a past transient ischemic attack or stroke (29). Current cigarette smoking and alcohol consumption of 15 g or more per day in the past year were defined accordingly (30). Neuroimaging was independently reviewed by two seasoned neurologists, with disagreements resolved through group discussions. Due to the heterogeneity in NIHSS and GCS score assessments in medical records, a third-party assessment team, blinded to the study group and treatment assignments, conducted a centralized review based on the available records. Any discrepancies in results led to intervention by a third assessor. A trained nurse or neurologist assessed the patients’ swallowing function using the Kubota Water Swallowing Test (31). Dysphagia was defined as a score of 3 or higher, or if the patient was unable to cooperate due to impaired consciousness. Based on patient information, the risk of stroke-associated pneumonia was calculated using the Intracerebral Hemorrhage-Associated Pneumonia Score (ICH-APS) (21).

### Malnutrition screening tools

The NRS-2002, CONUT, and PNI were used to assess patients’ nutritional status. The NRS-2002 was applied by trained nurses to evaluate the nutritional status of patients upon admission. The NRS-2002 score ranges from 0 to 7 and includes three components: (1) disease severity (indicating increased nutritional needs), ranging from 0 to 3 based on comorbidities and medical history; (2) nutritional impairment, based on BMI, body weight, and food intake, with a score range of 0 to 3; and (3) age, with one point assigned for patients aged 70 years or older. Patients with NRS-2002 score of 3 or greater are classified as being at risk of malnutrition (23). The CONUT is calculated using three indicators: serum albumin level, peripheral blood lymphocyte count, and total cholesterol level. Points are assigned based on laboratory results: (1) Serum albumin: ≥ 35 g/L = 0 points, 30–34 g/L = 2 points, 25–29 g/L = 4 points, < 25 g/L = 6 points; (2) Lymphocyte count: ≥ 1.6 × 10^9^/L = 0 points, 1.2–1.6 × 10^9^/L = 1 point, 0.8–1.1 × 10^9^/L = 2 points, < 0.8 × 10^9^/L = 3 points; (3) Total cholesterol: ≥ 180 mg/dL = 0 points, 140–179 mg/dL = 1 point, 100–139 mg/dL = 2 points, < 100 mg/dL = 3 points. The total score (0–12) reflects nutritional status, with higher scores indicating worse nutrition (32). The PNI is calculated by adding the serum albumin level to 5 times the peripheral blood lymphocyte count (33).

### Statistical analysis

Continuous variables were presented as medians with interquartile ranges (IQRs). Categorical variables were expressed as numbers and percentages. Comparisons between two groups for continuous variables were made using the Mann–Whitney U-test. For categorical variables, differences between the groups were analyzed using Fisher’s exact test or the Chi-square (χ^2^) test, as appropriate. Univariate logistic regression was used to identify risk factors for SICH-associated pneumonia. Stepwise multivariate logistic regression, adjusting for variables with *p* < 0.05 in the univariate analysis, was then applied to further examine the relationship between the NRS-2002 score and pneumonia in patients with SICH. SHAP (SHapley Additive exPlanations) is used to assess the contribution of each variable to the risk of pneumonia in patients with SICH. Receiver operating characteristic (ROC) analyses were conducted to evaluate the predictive performance of various nutritional scores and to externally validate the well-established ICH-APS model. Restricted cubic splines were utilized to explore the linear relationship between the NRS-2002 score and SICH-associated pneumonia. Subgroup analysis was performed to investigate potential moderating variables in the association between the NRS-2002 score and SICH-associated pneumonia. All statistical tests were two-tailed, with a significance level set at *p* < 0.05. Data analysis was conducted using PASS version 15, R version 4.0.4, SPSS version 26, and GraphPad Prism 9.0.

## Results

### Baseline patient characteristics

Based on the sample size calculation result, a minimum of 95 pneumonia patients and 95 non-pneumonia patients were required to achieve reliable results. A total of 697 patients with spontaneous intracerebral hemorrhage were initially considered for inclusion. Among them, 65 patients were excluded due to intracerebral hemorrhage resulting from brain tumors, aneurysms, arteriovenous malformations, cavernous hemangiomas, or traumatic brain injuries; 189 patients were excluded because of mechanical ventilation; 6 patients were excluded for having pre-existing pulmonary infection prior to admission; 5 patients were excluded due to a hospital stay of less than 24 h; and 28 patients were excluded due to incomplete data. Ultimately, 404 patients met the inclusion criteria, comprising 97 with pneumonia and 307 without pneumonia, meeting the minimum sample size requirement. Baseline characteristics of these patients were presented in Table 1. The average age of the included patients was 65.2 years, and 280 (69.3%) were male. Of these, 155 had an NRS-2002 score ≥ 3. These patients were older, had a lower BMI, were more likely to have comorbidities, and exhibited lower levels of blood lipids and blood cell counts compared to those with an NRS-2002 score < 3.

**Table 1:** Baseline characteristics of patients with spontaneous intracerebral hemorrhage: NRS-2002 ≥ 3 vs. NRS-2002 < 3.

Variables	All patients (*n* = 404)	NRS-2002 ≥ 3 (*n* = 155)	NRS-2002 < 3 (*n* = 249)	*p-*value
Demographic data				
Age (years), median (IQR)	66 (55,76)	77 (72, 83)	58 (51, 66)	<0.001
Sex, male, *n* (%)	280 (69.3%)	101 (65.2%)	179 (71.9%)	0.154
Weight (kg), median (IQR)	65.00 (56.00,72.00)	60.00 (52.50, 68.00)	68.00 (59.00, 76.00)	<0.001
Height (cm), median (IQR)	165.00 (158.00,170.00)	162.00 (156.00, 170.00)	167.00 (160.00, 170.00)	0.002
BMI, median (IQR)	23.90 (21.72,26.30)	23.01 (20.40, 24.77)	24.49 (22.06, 26.96)	<0.001
Vascular risk factors, *n* (%)				
Hypertension	321 (79.5%)	123 (79.4%)	198 (79.5%)	0.969
Diabetes mellitus	54 (13.4%)	20 (12.9%)	34 (13.7%)	0.829
Ischemic heart disease	24 (5.9%)	18 (11.6%)	6 (2.4%)	<0.001
History of stroke/TIA	66 (16.3%)	29 (18.7%)	37 (14.9%)	0.309
Current smoke	117 (29.0%)	29 (18.7%)	88 (35.3%)	<0.001
Excess alcohol consumption	98 (24.3%)	27 (17.4%)	71 (28.5%)	0.011
Comorbidities, *n* (%)				
COPD	24 (5.9%)	16 (10.3%)	8 (3.2%)	0.003
Dementia	20 (5.0%)	12 (7.7%)	8 (3.2%)	0.041
Cancer	22 (5.4%)	14 (9.0%)	8 (3.2%)	0.012
Baseline data				
Admission NIHSS score, median (IQR)	5.00 (2.00,11.00)	10.00 (3.00, 17.50)	4.00 (2.00, 8.00)	<0.001
Admission GCS score, median (IQR)	15.00 (13.00,15.00)	13.00 (9.00, 15.00)	15.00 (15.00, 15.00)	<0.001
Pre-stroke dependence (mRS ≥ 3), *n* (%)	17 (4.2%)	7 (4.5%)	10 (4.0%)	0.808
Dysphagia, *n* (%)	159 (39.4%)	96 (61.9%)	63 (25.3%)	<0.001
Infratentorial location, *n* (%)	56 (13.9%)	26 (16.8%)	30 (12.0%)	0.181
Extension into ventricles, *n* (%)	103 (25.5%)	53 (34.2%)	50 (20.1%)	0.002
Hemorrhage volume (ml), median (IQR)	8.05 (2.68,17.92)	10.70 (3.45, 27.65)	6.90 (2.50, 13.00)	<0.001
Antacid use, *n* (%)	366 (90.6%)	141 (91.0%)	225 (90.4%)	0.839
Laboratory data, median (IQR)				
WBC (*10^9^/L)	7.31 (5.75,9.73)	7.12 (5.61, 10.05)	7.40 (5.82, 9.50)	0.553
Neutrophil (*10^9^/L)	4.88 (3.61,6.96)	4.54 (3.44, 7.62)	4.96 (3.76, 6.71)	0.773
Lymphocyte (*10^9^/L)	1.44 (1.00,2.08)	1.28 (0.84, 2.04)	1.55 (1.11, 2.08)	0.002
RBC (*10^12^/L)	4.54 (4.08,4.92)	4.28 (3.87, 4.67)	4.65 (4.32, 5.01)	<0.001
Hemoglobin (g/L)	139.00 (128.00,149.00)	131.00 (122.00, 143.00)	143.00 (132.00, 152.00)	<0.001
Platelet (*10^9^/L)	202.00 (162.00,242.25)	187.00 (147.00, 234.50)	211.00 (174.00, 244.00)	<0.001
Serum creatinine (μmol/L)	68.50 (58.00,84.00)	68.00 (57.00, 84.00)	69.00 (59.00, 83.00)	0.908
Admission blood sugar (mmol/L)	5.55 (4.90,6.61)	5.71 (4.92, 7.03)	5.41 (4.89, 6.32)	0.065
Albumin (g/L)	38.30 (35.90,40.20)	36.90 (35.05, 39.20)	39.10 (36.70, 40.60)	<0.001
TG (mmol/L)	0.96 (0.68,1.48)	0.80 (0.58, 1.06)	1.07 (0.77, 1.65)	<0.001
LDL (mmol/L)	2.35 (1.83,2.98)	2.10 (1.63, 2.81)	2.53 (2.05, 3.09)	<0.001
HDL (mmol/L)	1.16 (0.98,1.36)	1.19 (1.02, 1.41)	1.15 (0.94, 1.34)	0.025
TC (mmol/L)	4.04 (3.48,4.67)	3.73 (3.24, 4.54)	4.20 (3.64, 4.77)	<0.001
Scoring scale				
CONUT score, median (IQR)	2.00 (1.00,4.00)	3.00 (1.00, 5.00)	2.00 (1.00, 3.00)	<0.001
PNI score, median (IQR)	45.73 (42.12,50.21)	44.00 (40.20, 48.10)	46.90 (43.50, 50.45)	<0.001
ICH-APS-A, median (IQR)	5.00 (2.00,8.00)	8.00 (5.00, 11.50)	3.00 (2.00, 5.00)	<0.001
ICH-APS-B, median (IQR)	5.00 (2.00,9.00)	9.00 (5.00, 12.00)	3.00 (2.00, 6.00)	<0.001
SAP, *n* (%)	97 (24.0%)	72 (46.5%)	25 (10.0%)	<0.001
Length of hospital stay (d), median (IQR)	10.00 (9.00,13.00)	11.00 (9.00, 14.00)	10.00 (8.00, 12.00)	<0.001

NRS-2002, nutritional risk screening-2002; BMI, body mass index; COPD, chronic obstructive pulmonary disease; NIHSS, national institutes of health stroke scale; GCS, Glasgow coma scale; mRS, modified Rankin scale; WBC, white blood cell; RBC, red blood cell; TG, triglycerides; LDL, low-density lipoprotein cholesterol; HDL, high-density lipoprotein cholesterol; TC, total cholesterol; CINUT, controlling nutritional status; PNI, prognostic nutritional index; ICH-APS, intracerebral hemorrhage-associated pneumonia score; SAP, stroke-associated pneumonia.

### Relationship between NRS-2002 score and SICH-associated pneumonia

**Figure 1:**
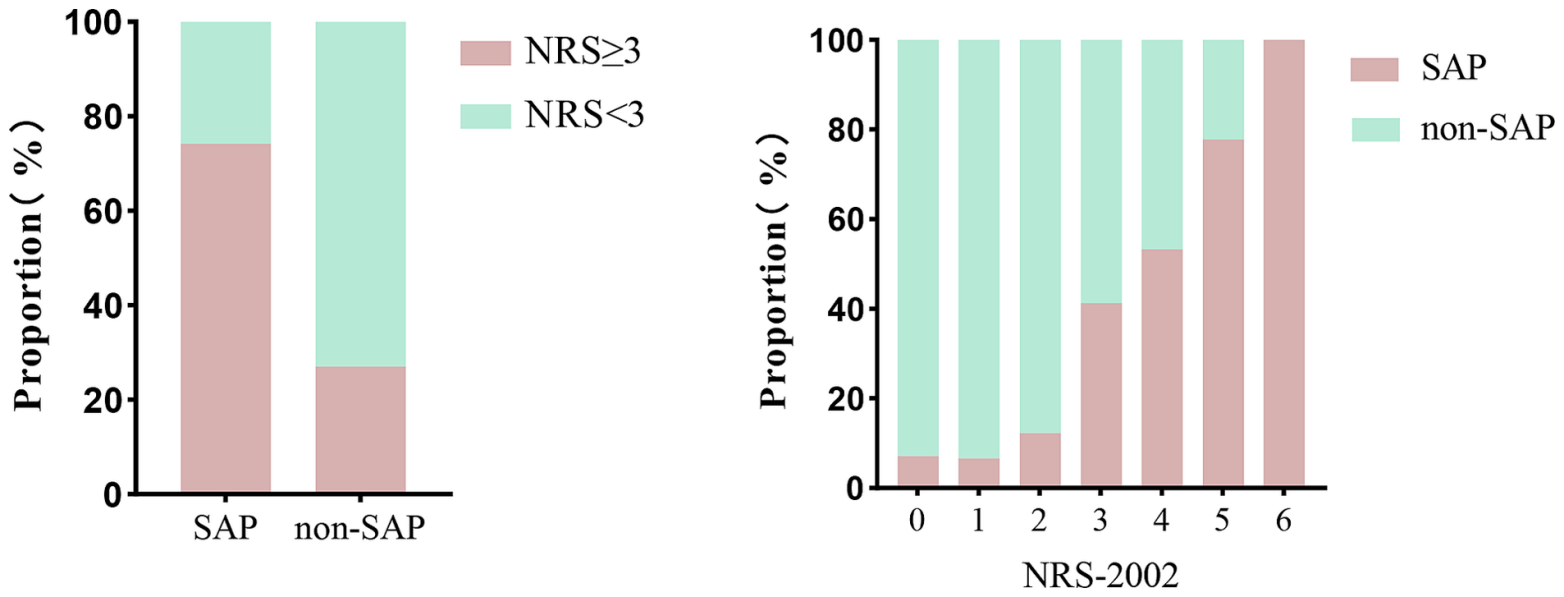
Probability distribution histogram illustrated the association of NRS-2002 and SAP. The proportion of patients with malnutrition (NRS-2002 ≥ 3) in SAP is higher than that in non-SAP (74.2% vs. 27.0%, *p* < 0.001). An increase in the NRS-2002 score is associated with a higher risk of pneumonia (P-trend <0.001). NRS-2002, nutritional risk screening-2002; SAP, stroke-associated pneumonia.

**Figure 2:**
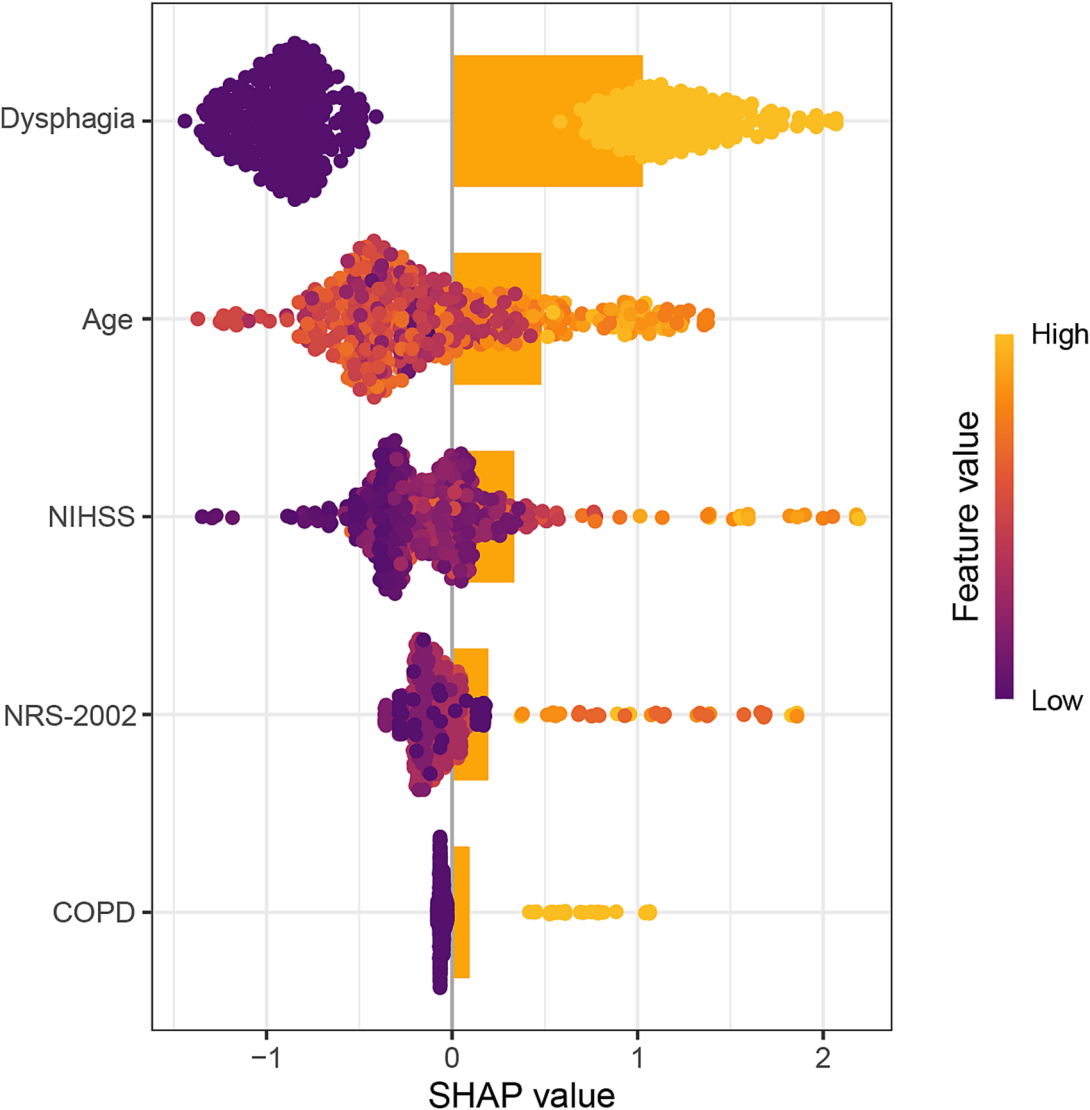
SHAP analysis of the contribution of each risk factor in predicting spontaneous intracerebral hemorrhage-related pneumonia. SHAP, SHapley Additive exPlanations; NRS-2002, nutritional risk screening-2002; NIHSS, national institutes of health stroke scale; COPD, chronic obstructive pulmonary disease.

**Figure 3:**
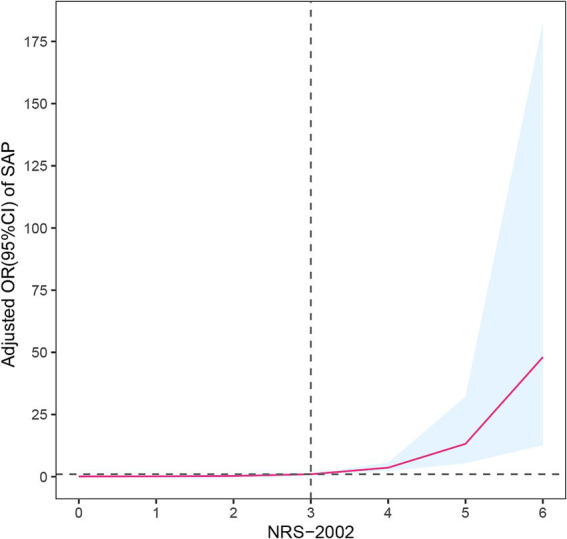
Restricted cubic spline model illustrated the association between NRS-2002 and SAP (P overall = 0.013, P non-linear = 0.151). The model accounts for confounding factors, including age, sex, NIHSS score, COPD, and dysphagia. The incidence of SAP increased significantly with higher NRS-2002 score, especially when the score was ≥3. NRS-2002, nutritional risk screening-2002; SAP, stroke-associated pneumonia; NIHSS, national institutes of health stroke scale; COPD, chronic obstructive pulmonary disease.

Compared with non-pneumonia patients, those with pneumonia had a significantly higher proportion of NRS-2002 scores ≥3 (74.2% vs. 27.0%, *p* < 0.001). Moreover, there was a clear upward trend in the proportion of pneumonia patients with increasing NRS-2002 score (*P*-trend <0.001) (Figure 1). In multivariate logistic regression analysis, after adjusting for variables with *p* < 0.05 in the univariate analysis, the NRS-2002 score remained significantly associated with an increased risk of SAP (OR: 1.575, 95% CI: 1.134–2.186, *p* = 0.007). Additionally, other factors such as age (OR: 1.045, 95% CI: 1.017–1.073, *p* = 0.002), chronic obstructive pulmonary disease (COPD) (OR: 3.734, 95% CI: 1.198–11.643, *p* = 0.023), dysphagia (OR: 6.407, 95% CI: 3.023–13.582, *p* < 0.001), and NIHSS score (OR: 1.075, 95% CI: 1.031–1.120, *p* < 0.001) were also identified as significant risk factors for SAP (Table 2). The SHAP plot shows the importance of each feature (variables with *p* < 0.05 in multivariate logistic regression) in predicting stroke-associated pneumonia in patients with SICH (Figure 2). NRS-2002 is the fourth most significant positive contributor, while dysphagia is the strongest positive contributor. Restricted cubic spline (RCS) analysis showed that the incidence of SAP increased notably with higher NRS-2002 scores when the score was ≥3. This relationship became more pronounced as the NRS-2002 score increased (Figure 3).

**Table 2:** Logistic regression analysis for predicting SAP in patients with spontaneous intracerebral hemorrhage.

Variables	Univariable logistic analysis		Multivariable logistic analysis	
	OR (95%CI)	*p*-value	OR (95%CI)	*p*-value
Demographic data				
Age (years)	1.075 (1.053, 1.097)	<0.001	1.045 (1.017, 1.073)	0.002
Sex, male	0.986 (0.601, 1.616)	0.954		
Weight (kg)	0.964 (0.946, 0.982)	<0.001		
Height (cm)	0.954 (0.927, 0.980)	<0.001		
BMI	0.912 (0.858, 0.971)	0.004		
Vascular risk factors				
Hypertension	1.289 (0.714, 2.325)	0.399		
Diabetes mellitus	0.785 (0.388, 1.590)	0.502		
Ischemic heart disease	1.635 (0.677, 3.946)	0.274		
History of stroke/TIA	1.474 (0.822, 2.641)	0.193		
Current smoke	0.811 (0.484, 1.360)	0.428		
Excess alcohol consumption	0.647 (0.365, 1.145)	0.135		
Comorbidities				
COPD	5.010 (2.147, 11.688)	<0.001	3.734 (1.198, 11.643)	0.023
Dementia	2.210 (0.876, 5.575)	0.093		
Cancer	2.313 (0.957, 5.591)	0.063		
Baseline data				
Admission NRS-2002 score	2.823 (2.122, 3.756)	<0.001	1.575 (1.134, 2.186)	0.007
Admission NIHSS score	1.160 (1.119, 1.202)	<0.001	1.075 (1.031, 1.120)	<0.001
Admission GCS score	0.695 (0.635, 0.762)	<0.001		
Pre-stroke dependence (mRS ≥ 3)	1.336 (0.459, 3.892)	0.595		
Dysphagia	16.329 (8.891, 29.989)	<0.001	6.407 (3.023, 13.582)	<0.001
Infratentorial location	1.319 (0.702, 2.478)	0.390		
Extension into ventricles	2.893 (1.773, 4.722)	<0.001		
Hemorrhage volume (ml)	1.041 (1.026, 1.056)	<0.001		
Antacid use	4.032 (1.212, 13.416)	0.023		
Laboratory data				
WBC (*10^9^/L)	1.038 (0.969, 1.112)	0.282		
Neutrophil (*10^9^/L)	1.052 (0.981, 1.128)	0.157		
Lymphocyte (*10^9^/L)	0.887 (0.677, 1.162)	0.385		
RBC (*10^12^/L)	0.527 (0.363, 0.765)	<0.001		
Hemoglobin (g/L)	0.973 (0.960, 0.987)	<0.001		
Platelet (*10^9^/L)	0.994 (0.990, 0.998)	0.004		
Serum creatinine (μmol/L)	1.002 (0.999, 1.004)	0.135		
Admission blood sugar (mmol/L)	1.175 (1.064, 1.298)	0.001		
Albumin (g/L)	0.900 (0.841, 0.963)	0.002		
TG (mmol/L)	0.363 (0.222, 0.593)	<0.001		
LDL (mmol/L)	0.721 (0.539, 0.965)	0.028		
HDL (mmol/L)	1.370 (0.660, 2.842)	0.398		
TC (mmol/L)	0.746 (0.569, 0.977)	0.033		

BMI, body mass index; COPD, chronic obstructive pulmonary disease; NRS-2002, nutrition risk screening-2002; NIHSS, national institutes of health stroke scale; GCS, Glasgow coma scale; mRS, modified Rankin scale; WBC, white blood cell; RBC, red blood cell; TG, triglycerides; LDL, low-density lipoprotein cholesterol; HDL, high-density lipoprotein cholesterol; TC, total cholesterol; SAP, stroke-associated pneumonia.

### Predictive value of NRS-2002 score on SICH-associated pneumonia

**Figure 4:**
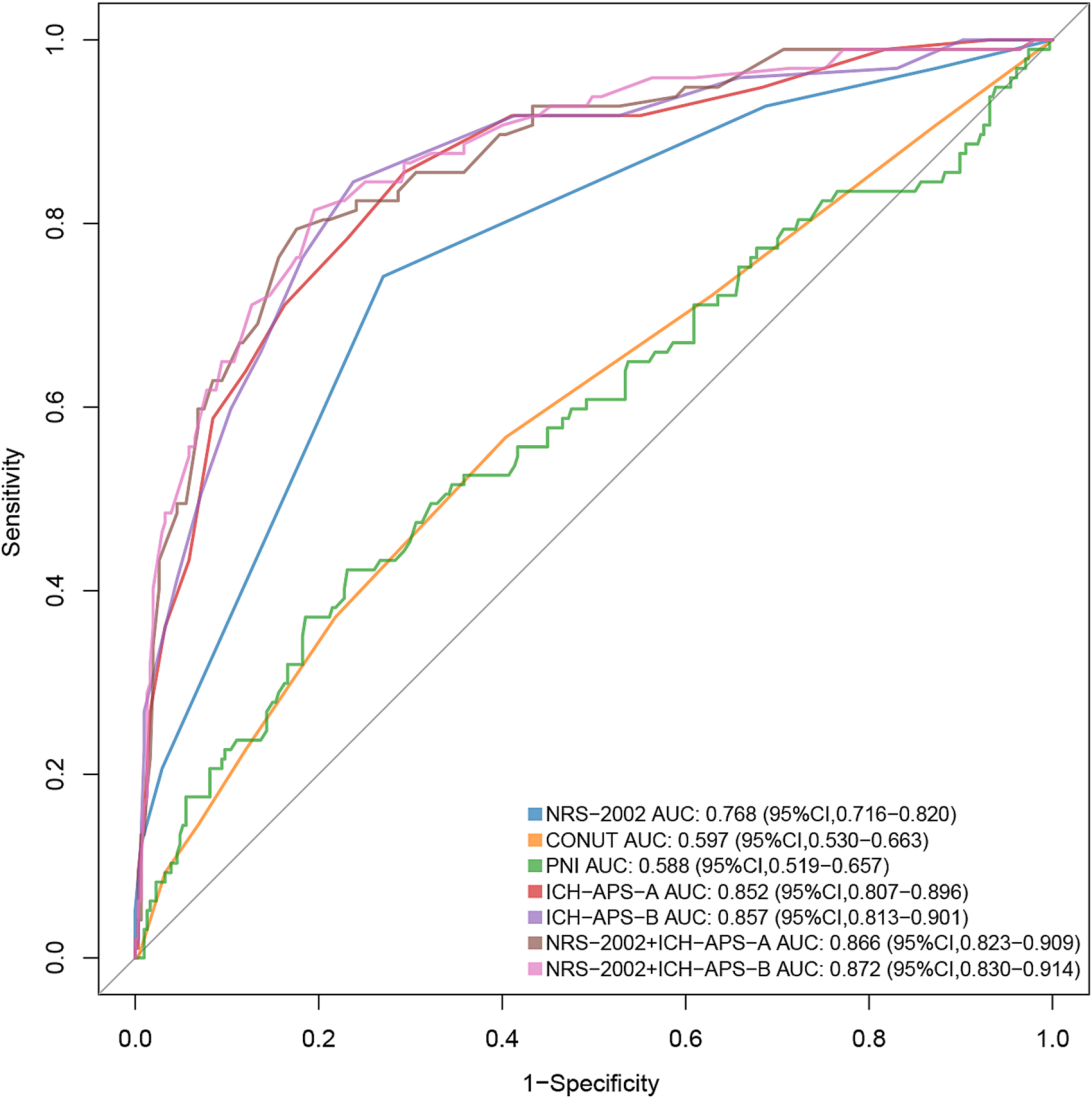
Comparison of AUCs between different predictive models. For predicting SAP, ICH-APS exhibited superior predictive performance compared to the nutritional assessment tools (*p* < 0.05). Among the nutritional scoring systems, NRS-2002 provided better predictive value than PNI and CONUT (*p* < 0.05). AUC, area under the curve; SAP, stroke-associated pneumonia; ICH-APS, intracerebral hemorrhage-associated pneumonia score; NRS-2002, nutritional risk screening-2002; PNI, prognostic nutritional index; CONUT, Controlling nutritional status.

Figure 4 presented the predictive performance of the NRS-2002 score for SAP using AUC-ROC (Area Under the Receiver Operating Characteristic Curve) analysis (AUC: 0.768, 95% CI: 0.716–0.820). The NRS-2002 score demonstrated superior predictive capability for SAP compared to the PNI (AUC: 0.588, 95% CI: 0.519–0.657) and the CONUT score (AUC: 0.597, 95% CI: 0.530–0.663) (*p* < 0.01). External validation of the classic ICH-APS-A (AUC: 0.852, 95% CI: 0.807–0.896) and ICH-APS-B (AUC: 0.857, 95% CI: 0.813–0.901) models showed better predictive performance than the NRS-2002 score (*p* < 0.01). Incorporating the NRS-2002 score into the ICH-APS-A and ICH-APS-B models slightly increased the AUC, but this improvement was not statistically significant (*p* > 0.05). Table 3 summarized the optimal cut-off points, specificity, sensitivity, positive predictive value, and negative predictive value for each scoring system.

**Table 3:** Comparison of evaluation metrics across different models in predicting the SAP.

	AUC (95% CI)	Cutoff point	Specificity	Sensitivity	PPV	NPV
NRS-2002	0.768 (0716, 0.820)	2.5	0.730	0.742	0.465	0.900
CONUT	0.597 (0.530, 0.663)	2.5	0.596	0.567	0.307	0.813
PNI	0.588 (0.519, 0.657)	42.65	0.769	0.423	0.366	0.808
ICH-APS-A	0.852 (0.807, 0.896)	5.5	0.707	0.856	0.480	0.934
ICH-APS-B	0.857 (0.813, 0.901)	6.5	0.762	0.845	0.529	0.940

AUC, area under the curve; SAP, stroke-associated pneumonia; ICH-APS, intracerebral hemorrhage-associated pneumonia score; NRS-2002, nutritional risk screening-2002; PNI, prognostic nutritional index; CONUT, controlling nutritional status; PPV, positive predictive value; NPV, negative predictive value.

### Subgroup analysis

**Figure 5:**
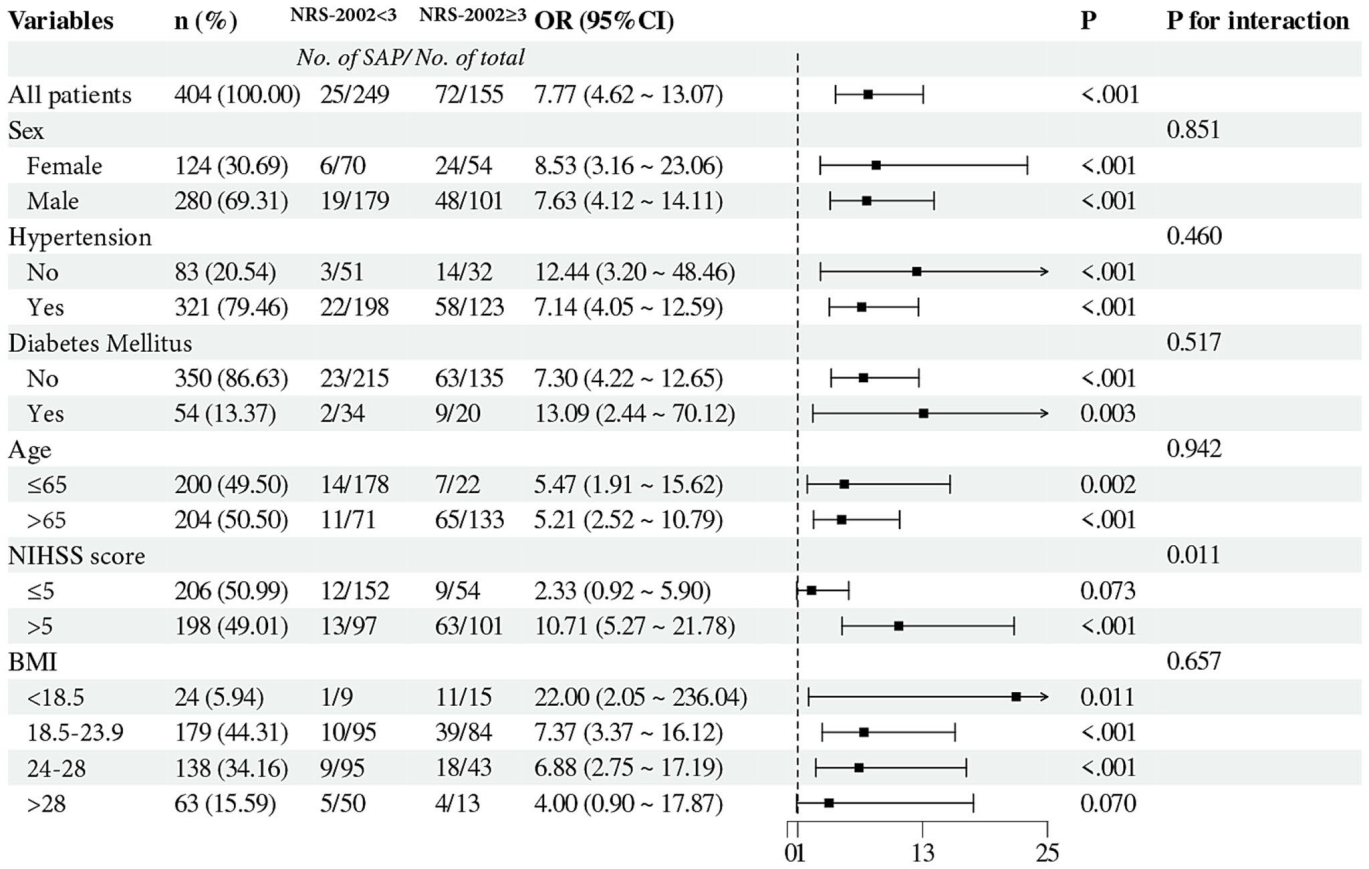
Subgroup analysis evaluating the interaction effect of NRS-2002 on the incidence of SAP across varied subgroups. The forest plot indicated that the NIHSS score might moderate the relationship between NRS-2002 and SAP occurrence (P for interaction = 0.011). NRS-2002, nutritional risk screening-2002; SAP, stroke-associated pneumonia; NIHSS, national institutes of health stroke scale; BMI, body mass index.

A subgroup analysis stratified patients by age, sex, BMI, NIHSS score, hypertension, and diabetes (Figure 5). The results demonstrated that an NRS-2002 score ≥ 3 was associated with a higher likelihood of SAP in patients with a BMI <18.5 (OR: 22.00, 95% CI: 2.05–236.04, *p* = 0.011), NIHSS score > 5 (OR: 10.71, 95% CI: 5.27–21.78, *p* < 0.001), absence of hypertension (OR: 12.44, 95% CI: 3.20–48.46, *p* < 0.001), and diabetes (OR: 13.09, 95% CI: 2.44–70.12, *p* = 0.003). The severity of stroke might influence the relationship between the NRS-2002 score and SAP (*P* for interaction =0.011).

## Discussion

Studies have demonstrated that the NRS-2002 score was linked to the short-term and long-term prognosis of hemorrhagic stroke patients (34). However, few studies have focused on the connection between the NRS-2002 score and pneumonia related to SICH. To the best of our knowledge, this was the first study to explore the association between the NRS-2002 score and SICH-related pneumonia. Our results indicated that higher NRS-2002 score at the time of admission was significantly associated with an increased risk of developing SICH-related pneumonia. These findings underscored the pivotal role of nutritional status in the acute-phase management of spontaneous intracerebral hemorrhage.

In our study, 38.4% of patients with SICH were identified as malnourished, aligning with prior findings on nutritional status in cerebral infarction patients assessed using the NRS-2002 score (35). Malnourished patients in our study were typically older, had lower BMI, and exhibited more comorbidities. This trend might be attributed to the emphasis of the NRS-2002 scoring criteria. However, when alternative nutritional assessment tools were applied to stroke patients, malnourished individuals consistently displayed similar baseline characteristics (14), indicating potential concordance among different scoring systems. Blood lipid levels differ significantly between malnourished and healthy populations in our study. The Chinese Visceral Adiposity Index (CVAI), an indicator of visceral fat in the Chinese population, is strongly associated with stroke incidence (36). Unlike the PNI and CONUT scores, which assess nutritional status from immune and inflammatory perspectives, CVAI reflects nutritional status through blood lipid profiles. Its potential link to stroke-associated pneumonia warrants further investigation. Furthermore, our study observed lower levels of immune and blood cells in malnourished patients, providing evidence to support the use of immune and inflammatory indicators in evaluating nutritional status. The ICH-APS is a recognized predictor of pneumonia in patients with SICH and is recommended by the Chinese clinical management guidelines for intracerebral hemorrhage to evaluate the risk of pneumonia. In malnourished patients, ICH-APS scores were significantly higher, which further validated the use of the NRS-2002 score in assessing pneumonia risk in patients with SICH. Interestingly, the study found a lower proportion of smokers and alcohol consumers among malnourished patients. We believe this might be due to our definition of smoking and drinking as current smoking and excessive alcohol consumption. Malnourished patients had comorbid conditions like COPD and ischemic heart disease in our study, and in real-world situations, these individuals tend to quit smoking and reduce alcohol intake.

Consistent with earlier research (37), our study based on the NRS-2002 score showed that malnutrition increases the risk of pneumonia related to SICH. Malnutrition leads to immune suppression, resulting in an increased incidence of pneumonia. Malnutrition compromises immune function through various mechanisms. It reduces T-cell counts, particularly CD4+ T cells with interleukin-2 receptors and CD3+ and CD25+ T cells, leading to impaired cell-mediated immunity (38). Furthermore, malnutrition affects phagocyte function, diminishing the body’s ability to clear pathogens, and disrupts complement synthesis and activity, weakening antiviral defenses (39). Additionally, malnutrition impairs muscle tissue repair and energy supply, leading to respiratory muscle weakness or even atrophy. This results in difficulty coughing up sputum and breathing, further increasing the risk of pneumonia (40, 41). In our study, we observed that advanced age increased the risk of SICH-associated pneumonia. Since age was a component of the NRS-2002 score, we employed stepwise regression to mitigate the effects of collinearity. Despite this adjustment, age continued to show a significant association with an increased risk of pneumonia in patients with SICH. Many previous studies have identified a relationship between age and stroke-associated pneumonia (3, 21, 42). In the ICH-APS scoring model, age is positively associated with the risk of developing SAP, with the risk increasing by 4% for each additional year (21). Similarly, another study on pneumonia following SICH reported a comparable positive correlation between age and SAP risk (3), with the same odds ratio found in the ICH-APS scoring model. Our study also yielded a similar OR of 1.045 (95% CI: 1.017–1.073). We hypothesize that age-related nutritional risks may significantly contribute to this association. Additionally, aging leads to a decline in both cellular and humoral immunity, weakening the body’s ability to effectively combat pathogens (43, 44). This compromised nutrition and immune defense allows pathogens to proliferate in the respiratory tract, increasing the risk of lung infections. In the AIS-ASP model, COPD is significantly associated with the development of stroke-associated pneumonia (SAP) following IS (adjusted OR = 1.67, 95% CI: 1.09–2.55) (20). This correlation is even stronger in patients with SICH. COPD patients have more than a fivefold higher risk of developing SAP after ICH compared to non-COPD patients in the ICH-APS model (21). Consistent with previous studies (3, 21, 42), our findings also indicate that COPD patients are at a higher risk of developing SAP (adjusted OR = 3.734, 95% CI: 1.198–11.643). COPD is characterized by chronic bronchitis, emphysema, and other lung pathologies (45), leading to chronic inflammation and structural changes in the airways (46, 47). In the context of intracerebral hemorrhage, immune suppression exacerbates these existing respiratory complications, further elevating the risk of lung infections (5). Dysphagia has the greatest impact on the weight coefficient of pneumonia for SICH in ICH-LR2S2 score (3). In our study, dysphagia also showed the highest odds ratio, indicating that it was the most significant risk factor for SAP. This could be due to the increased risk of aspiration pneumonia caused by swallowing difficulties (48). Previous studies have nearly always identified an association between NIHSS score and SAP when NIHSS score was included in the analysis (3, 21, 42), and our study aligned with this finding. We suggest that future research investigate the association between specific NIHSS score components and stroke-associated pneumonia, with a particular focus on dysarthria and level of consciousness, as these may play a more significant role than other components.

There are numerous nutritional risk assessment tools, each with its own criteria. They can generally be classified into two categories: objective and subjective. The former focuses on hematological, biochemical, and anthropometric evaluations, while subjective assessments tend to rely on nutritional assessment questionnaires and medical history collection (49). Through the DeLong test comparison of AUC, we observed that the NRS-2002 score demonstrates superior predictive performance for pneumonia associated with SICH compared to the CONUT and PNI nutritional scores. This may be due to the fact that markers such as albumin and lymphocytes are affected not only by nutritional status but also by liver and kidney function, as well as hematopoietic activity (50, 51). Since we did not exclude patients with mild hepatic and renal dysfunction during enrollment, this may impact the true reflection of nutritional status by PNI and CONUT. In contrast, the NRS2002 evaluates nutritional status from multiple dimensions, overcoming the limitations of relying on a single marker, and provides a more comprehensive and accurate assessment of a patient’s nutritional risk. To further minimize subjective scoring variability, we provided systematic training for the assessors in advance to reduce potential bias. We externally validated the ICH-APS model through AUC-ROC analysis, indicating that it has excellent predictive value for pneumonia associated with SICH. Considering that the ICH-APS model does not fully account for the impact of nutritional status on pneumonia development, we integrated the NRS-2002 with the ICH-APS model for analysis and found that it did not statistically improve its predictive effectiveness. Based on the SHAP results in the study, the contribution of NRS-2002 is smaller than that of other indicators such as dysphagia, age, and the NIHSS score in predicting SAP. These indicators are all part of the ICH-SAP model, making the predictive efficacy of NRS-2002 incomparable to that of the ICH-SAP model. Compared to the ICH-APS model, which requires the inclusion of multiple parameters for prediction, the NRS-2002 score is simpler and more practical, thus offering high clinical applicability. We further used sensitivity, specificity, negative predictive value, and positive predictive value to demonstrate the characteristics of each prediction model. The NRS-2002 score had a threshold of 2.5 for predicting SICH-associated pneumonia in our study, with a score of 3 or higher indicating nutritional risk according to the guidelines (23). This further confirms the association between malnutrition and SICH-associated pneumonia. Notably, all the models demonstrated good negative predictive values, but their positive predictive values were relatively low. Therefore, the primary utility of these models is to help exclude patients with pneumonia in clinical practice.

The subgroup analysis identified a stronger correlation between malnutrition and SICH-associated pneumonia in patients with diabetes and those without hypertension. In diabetic patients, insulin resistance contributes to relative insulin deficiency and impaired glucose utilization, increasing the risk of malnutrition (12). Among non-hypertensive patients, a higher prevalence of non-hypertensive intracerebral hemorrhage may impact nutritional risk and the incidence of pneumonia. Future research should investigate and compare the prevalence of malnutrition and pneumonia across patients with intracerebral hemorrhage of varying etiologies. RCS analysis revealed that the relationship between malnutrition and pneumonia strengthens as NRS-2002 scores increase in malnourished patients. Since BMI is a key component of the NRS-2002 tool (23), individuals with low BMI are more likely to experience severe malnutrition. Additionally, due to the reduced expression of immune factors, patients with low BMI have weaker immune resistance (52), which increases their susceptibility to various infections during hospitalization. Besides pneumonia, they are also more prone to urinary tract infections, skin and soft tissue infections, and other complications (53, 54), which contribute to a higher disease severity and subsequently lead to an increased NRS-2002 score. As a result, underweight patients with malnutrition face a substantially higher risk of pneumonia compared to those with normal weight, overweight, or obesity. A significant interaction was observed between NRS-2002 and NIHSS scores in predicting the occurrence of SICH-associated pneumonia. Neurological deficits have been identified as risk factors for malnutrition (3, 21, 42, 55). Compared to patients with mild stroke, those with moderate to severe stroke are at a higher risk of malnutrition. As nutritional risk increases, the relationship between pneumonia and malnutrition becomes more pronounced, leading to a greater risk of pneumonia in malnourished patients with moderate to severe stroke.

In this study, we demonstrated that the NRS2002-based nutritional score can effectively predict the risk of stroke-associated pneumonia during hospitalization in patients with spontaneous intracerebral hemorrhage at the time of admission. For patients identified as being at risk of malnutrition, early consultation with a dietitian and timely, appropriate nutritional support, along with improved oral care and respiratory management, can be provided to reduce the risk of pneumonia. The NRS-2002 score does not require additional diagnostic procedures, making it feasible for implementation in both developing and developed countries. However, certain parameters in the score, such as disease severity, rely on the evaluator’s clinical judgment. Therefore, systematic and standardized training for evaluators is crucial. This study has several limitations. First, as a single-center retrospective study, it might be subject to selection bias. A multicenter study is essential for future research. Second, inflammatory markers such as C-reactive protein and procalcitonin were not included (56, 57), hindering the ability to explore the relationship between these markers, malnutrition, and stroke-associated pneumonia. Lastly, the study only focused on the relationship between baseline nutritional status and stroke-associated pneumonia, without tracking changes in nutritional status over time.

## Conclusion

The NRS-2002 score is associated with stroke-related pneumonia in patients with spontaneous intracerebral hemorrhage. Patients with a nutritional risk (NRS-2002 ≥ 3) are at a higher risk of developing pneumonia. Compared to other nutritional assessment tools, the NRS-2002 score shows greater predictive effectiveness.

## Data Availability

The raw data supporting the conclusions of this article will be made available by the authors, without undue reservation.
